# A Novel High‐Safety Filler with Potential Periosteal Enhancement: In Vivo Evaluation of Cross‐Linked Sodium Hyaluronate Gel with PVA Microspheres in Rat Models

**DOI:** 10.1111/jocd.70522

**Published:** 2025-10-29

**Authors:** Yuchen Dai, Xiaowen Liu, Zhen Li, Huanyun Niu, Shiwei Wang, Dongmei Tan

**Affiliations:** ^1^ Laboratory Animal Center Chongqing Medical University Chongqing China; ^2^ Bejing Engineering Lab of Neo‐Biodegradable Materials Beijing China

**Keywords:** angiogenesis, collagen fibers, hyaluronic acid, inflammatory response, periosteal thickening, polyvinyl alcohol, tissue filling

## Abstract

**Background:**

In recent years, the demand for sophisticated aesthetic solutions and the emergence of novel materials in cosmetic dermatology have increased substantially. However, these materials induce marked inflammatory reactions. To address this limitation, in this study, we developed a novel filler Cross‐Linked Sodium Hyaluronate Gel combined with Polyvinyl Alcohol Microspheres (PVA/HA filler) and systematically evaluated its effects on the periosteum and subcutaneous tissue in a rat model.

**Methods:**

Ultrasound, histopathology, and molecular biological assays were employed to systematically assess the shape retention of the PVA/HA filler and its biological responses at 1, 3, and 6 months in the periosteum and subcutaneous tissues.

**Results:**

Experimental data demonstrated that the PVA/HA filler exhibited excellent structural support, with no significant morphological changes observed over the 6‐month observation period. Notably, the filler effectively promoted periosteal angiogenesis and enhanced proliferation of collagen and elastic fibers. Furthermore, it maintained low inflammatory responses, even with increased microsphere concentrations in the PVA/HA formulation.

**Conclusions:**

The novel PVA/HA filler demonstrated strong structural support in both the periosteum and subcutaneous tissue. The filler provides enhanced periosteal angiogenesis, promotes periosteal thickening, and stimulates collagen fiber formation, while exhibiting minimal inflammation, high safety, and no risk of hyperplasia.

## 1 Introduction

Dermal aging is characterized by progressive degradation of the extracellular matrix and the concomitant quantitative and functional decline of fibroblasts. This pathophysiological progression can manifest clinically as rhytides, volumetric tissue loss, subcutaneous atrophy, and compromised connective tissue integrity [1]. In addition, aging causes reductions in cell vitality in the facial periosteum and progressive bone resorption, both of which are major factors in aging [2, 3]. In response to these challenges, various dermal fillers have been developed over the years to address soft tissue deficiencies. Notable examples include Poly‐L‐lactic Acid (PLLA), Calcium Hydroxylapatite (CaHA), and Polymethyl Methacrylate (PMMA), which are widely used for their ability to provide structural support with minimal invasiveness [4, 5]. These materials aim to offer long‐term stability with minimal adverse effects, effectively addressing aesthetic concerns [6]. However, these materials can induce inflammation post‐implantation, often causing local swelling and nodules. There is a risk of damage to the periosteum [7]. The present study aimed to address these challenges and investigate potential solutions.

The Polyvinyl alcohol (PVA) is a water‐soluble polymer with excellent biocompatibility and hydrophilicity [8, 9]. Consequently, PVA has been widely used in the preparation of various biomedical materials, such as artificial joints, viscoelastic agents, and drug delivery systems [10, 11, 12, 13]. Research has shown that PVA enhances osteoblast adhesion and proliferation, upregulates the expression of osteoblast‐related gene expression, and significantly promotes in vitro osteogenesis, osteoinduction, and bone regeneration [14]. Additionally, PVA has been shown to influence periosteal activity [15]. Moreover, PVA‐based microspheres are associated with a lower inflammatory response than conventional microsphere fillers. When engineered into spherical architectures analogous to traditional formulations, they have enhanced safety profiles. The inherent inability of PVA microspheres to achieve immediate filling effects necessitates the selection of an optimized delivery system.

The hyaluronic acid (HA), a naturally occurring polysaccharide in human tissues, has seen widespread use in medical aesthetics due to its excellent biocompatibility and biodegradability [16]. This biomaterial not only provides immediate supportive effects but also allows for slower degradation and extended therapeutic efficacy through cross‐linking technology. HA exhibits excellent suspension capacity, enabling its widespread integration with various microspheres of diverse physicochemical properties and functional components and facilitating the development of customized fillers [17].

Leveraging these advantages, this study successfully developed a novel filler by optimizing the manufacturing process and using cross‐linked HA to suspend PVA microspheres. This composite formulation is designed to achieve dual therapeutic goals: it can act on the subperiosteal layer by providing immediate morphological stabilization and a sustained supportive effect. This stands in sharp contrast to current commercial PVA/HA products, which contain hydroxypropyl methylcellulose (HPMC) and lack a clear indication for subperiosteal application. This composite formulation was designed to achieve two therapeutic objectives: immediate morphological stabilization and sustained support through maintained periosteal positioning of PVA microspheres. The PVA/HA filler was administered to the forehead periosteum and subcutaneous tissue in Sprague–Dawley rats to explore its effects in vivo. The evaluation focused on ultrasound observations and histopathological analysis to assess the filler's effects on the periosteum and subcutaneous tissue.

## 2 Materials and Methods

### 2.1 Materials

The PVA/HA filler (30 mg/mL PVA, 10 mg/mL HA), Triple PVA/HA filler (90 mg/mL PVA, 10 mg/mL HA), and cross‐linked HA (10 mg/mL HA). The Hematoxylin & Eosin (H&E) staining solution (G1003), Masson staining solution (G1006), Movat staining solution (G1042, S8020, G1027), CD31 primary antibodies (GB11063‐1), and IL‐6 primary antibodies (GB11117) were purchased from Servicebio Technology Co. Ltd. (Wuhan, China).

### 2.2 Animals and Experimental Design

A total of 36 male Sprague–Dawley rats (8 weeks old) were maintained under optimal conditions at a temperature of 23°C ± 3°C and relative humidity of 40%–70% on a 12‐h light/12‐h dark cycle and fed with food and water ad libitum. After 1 week of acclimatization, rats were randomly divided into the control group (implanted with HA filler), Single PVA group (implanted with PVA/HA filler), and Triple PVA group (implanted with Triple PVA/HA filler). The rats were anesthetized with xylazine hydrochloride injection. The fillers were implanted in the forehead and the back subcutaneous tissue at a volume of 0.5 mL per injection site.

### 2.3 Ultrasound Observation

A Vinno 6 lab ultrasound diagnostic instrument (X10‐23 L; Suzhou, China) was used in this experiment to detect the parietal bone and back filling area at 1, 3, and 6 months after filling. The provided ultrasound images were used for experimental analysis, including measurements of relevant structural dimensions and evaluation of tissue characteristics [18].

### 2.4 Histopathological Staining

At 1, 3, and 6 months post‐injection, three rats were randomly selected from each group; following anesthesia using xylazine hydrochloride injection, rats were euthanized via cervical dislocation. Injection sites and adjacent tissues were systematically harvested. The specimens were promptly fixed in 10% neutral‐buffered formalin. After decalcification of the bone within the samples, all tissues were embedded in paraffin. Using a microtome, the specimens were vertically sectioned along the longitudinal axis into 5‐μm thick slices. These slices were then subjected to hematoxylin and eosin (H&E), Masson's trichrome, and Movat–Russell modified pentachrome stainings. Subsequently, they were observed after scanning by slice scanners (Pannoramic DESK, 3DHISTECH, Budapest, Hungary). The inflammatory response in the vicinity of the injection site was graded in accordance with ISO 10993 evaluation criteria. Meanwhile, the collagen content in each injected area was quantified using Image J (Media Cybernetics Inc., Bethesda, MD, USA).

### 2.5 Fluorescence Immunohistochemistry

Immunofluorescence staining was performed to characterize the expression of platelet endothelial cell adhesion molecule‐1 (PECAM‐1, CD31) and interleukin 6 (IL‐6), and the fluorescence intensity was scanned using a slice scanner (Pannoramic DESK, 3DHISTECH, Budapest, Hungary).

### 2.6 Statistical Analysis

Data are expressed as the Mean ± SEM of at least three independent experiments. GraphPad Prism 8 (GraphPad Software, San Diego, CA, USA) was used for statistical analysis. Groups were compared using the two‐tailed Student's *t*‐test with a significance threshold of *p* < 0.05.

## 3 Results

### 3.1 Ultrasound Demonstrated Supporting Effects of PVA/HA Filler

**FIGURE 1:**
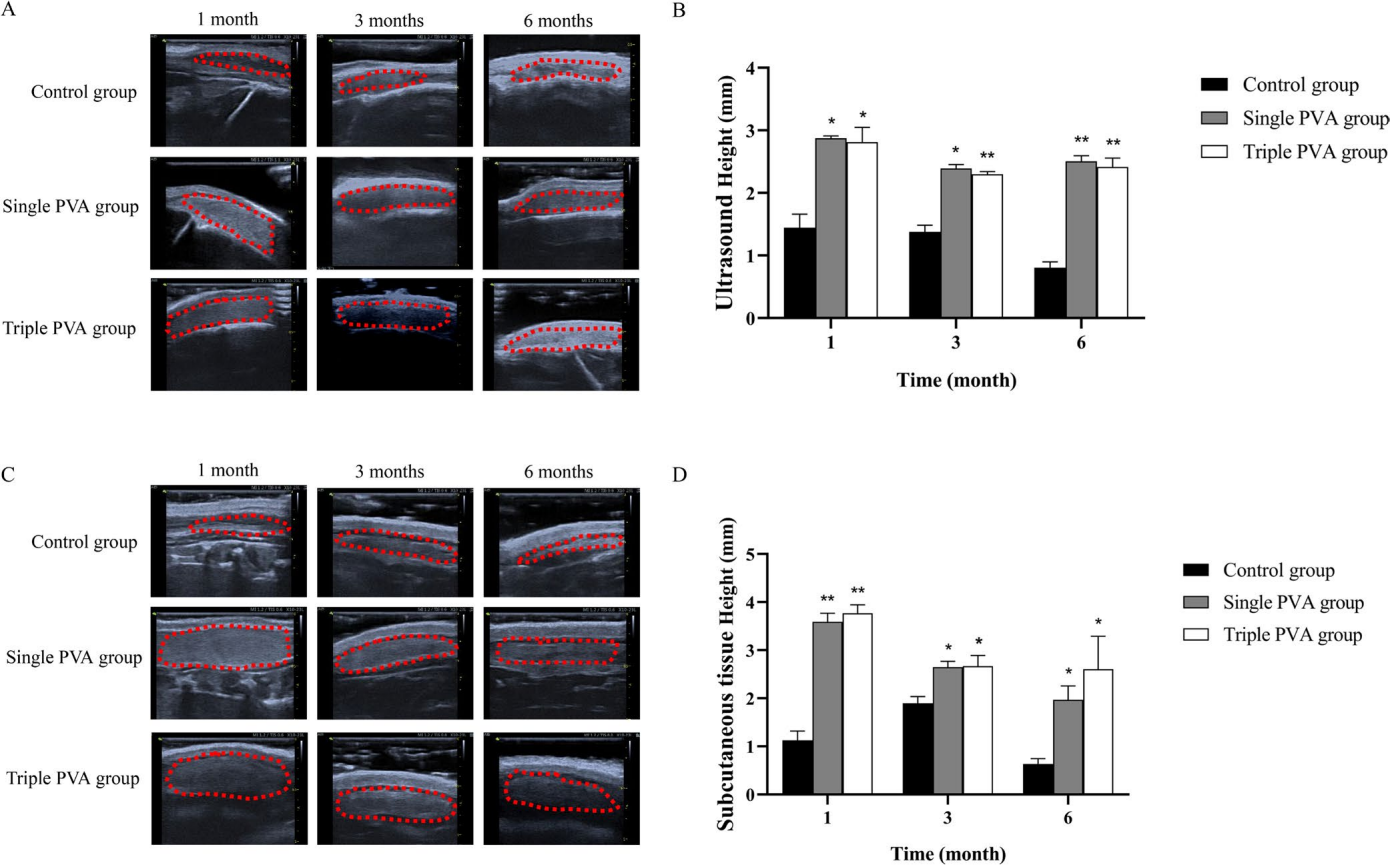
Ultrasound results demonstrating the supporting effect of HA/PVA filler. (A) Ultrasound images after sample injection upon the periosteum in each group. (B) Quantitative analysis of sample height at the periosteum in each group. (C) Ultrasound images after subcutaneous injection of samples in each group. (D) Quantitative analysis of sample height at the subcutaneous site in each group. *p < 0.05, **p < 0.01 vs. Control group, *n* = 3.

Ultrasound images of the forehead in rats showed that from 1 month to 6 months post‐implantation, the PVA/HA filler in the Single PVA group and Triple PVA groups maintained a relatively stable shape without significant deformation (Figure 1A). The sample height in the Single PVA group and Triple PVA group at corresponding time points were significantly higher than those in the Control group, which exhibited a decrease in sample height over the same period (Figure 1B).

Similarly, ultrasound images of subcutaneous sites on the back showed good support from the PVA under the skin at the 6‐month observation (Figure 1C). In contrast, the Control group, implanted with HA, showed a reduction in support height (Figure 1D).

### 3.2 The PVA/HA Filler Promoted Periosteal Thickening

**FIGURE 2:**
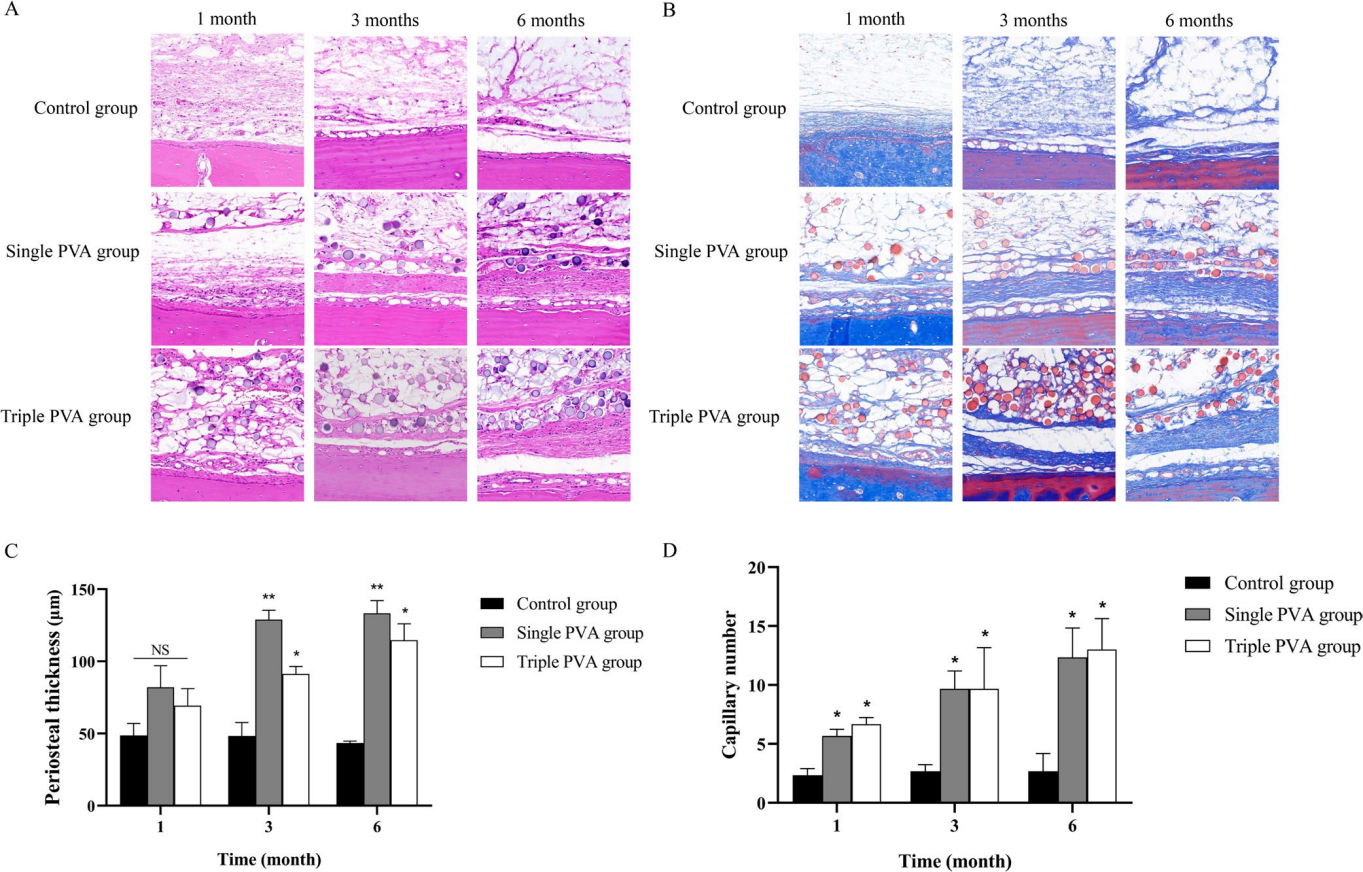
Histological results of periosteum in each group. (A) H&E staining images after sample injection upon the periosteum in each group (100×). (B) Masson staining images after sample injection upon the periosteum (100×). (C) Quantitative analysis of periosteal thickness in each group. (D) Quantitative analysis of capillary numbers at the periosteum in each group. *p < 0.05, **p < 0.01, NS (no significant difference) vs. Control group, *n* = 3.

The H&E and Masson staining demonstrated that PVA/HA filler injection upon the periosteum increased periosteal thickness over time, with PVA microspheres maintaining stable positioning and intact morphology. The Single PVA group and the Triple PVA group exhibited a gradual thickening trend from 1 to 6 months, while the Control group showed no significant changes (Figure 2A,B).

And in this study, both the Single and Triple PVA groups exhibited greater periosteal thickness and demonstrated significantly increased capillary density at the periosteal interface compared to the Control group across all observations (Figure 2C,D).

### 3.3 PVA/HA Filler Promoted Subcutaneous Fiber Growth With Only Slight Inflammatory Responses

**FIGURE 3:**
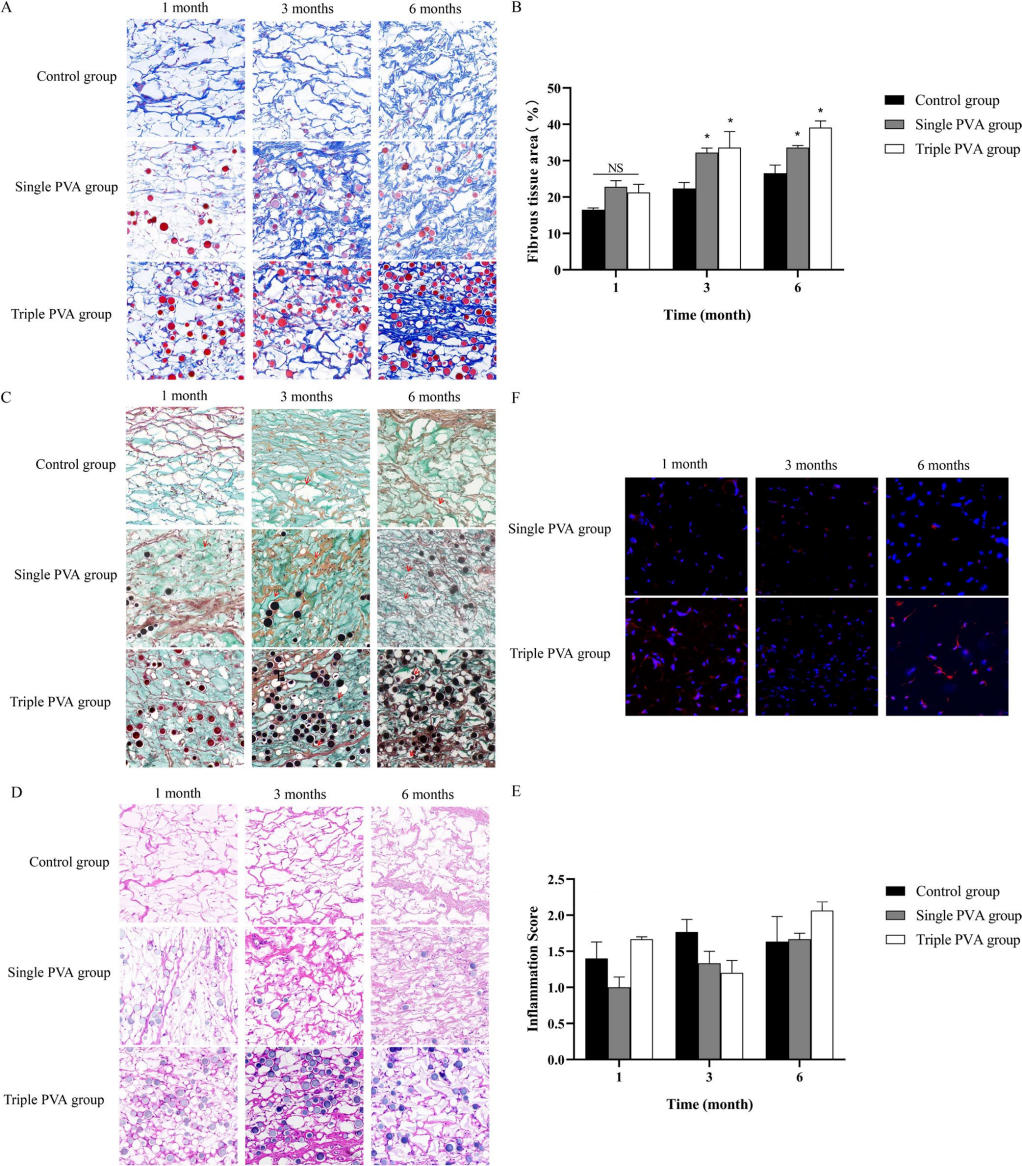
Histological results showing that PVA/HA filler promotes subcutaneous fiber growth. (A) Masson staining images showing collagen fibers after subcutaneous injection in each group (100×). (B) Quantitative analysis of subcutaneous collagen fibers in Masson sections of each group. (C) Movat staining images (elastic fibers indicated by arrows) after subcutaneous injection in each group (100×). (D) H&E staining images showing after subcutaneous injection of samples in each group (100×). (E) Quantitative analysis of inflammatory responses in H&E‐stained sections from the subcutaneous injection groups showed no significant differences among all groups. (F) The IL‐6 immunofluorescence images demonstrating inflammation levels in each group (200×). *p < 0.05, NS (no significant difference) vs. Control group, *n* = 3.

After subcutaneous injection, Masson staining showed new fibrous structures that appeared at 1 month, increased significantly by 3 months, and became dense and orderly by 6 months in the Single and Triple PVA groups. The Control group exhibited slower growth with sparse fibers (Figure 3A). Quantitative analysis revealed significantly less collagen in the Control group than in the Single and Triple PVA groups at all observations (Figure 3B). Movat staining showed that the elastic fiber density and length increased over time in the Single and Triple PVA groups, becoming robust by 6 months, but the Control group exhibited minimal changes (Figure 3C).

It is noteworthy that PVA/HA Filler effectively promoted subcutaneous fiber growth while eliciting merely minimal inflammatory responses, which were demonstrated in H&E staining (Figure 3D). At the 1‐month post‐administration observation, quantitative analysis of inflammatory responses showed mild scores within acceptable ranges for all groups. Both the Single and Triple PVA groups showed minor inflammatory cell infiltration. At 3 and 6 months, inflammatory scores showed slight fluctuations while remaining within acceptable ranges, with histological findings comparable to those at 1 month (Figure 3E). Additionally, immunofluorescence analysis of IL‐6 expression demonstrated low inflammatory factor activity in the Single PVA and Triple PVA groups (Figure 3F).

## 4 Discussion

This study demonstrated favorable performance regarding mechanical support when using a novel PVA/HA filler. Ultrasound revealed dimensional stability in the implantation area without significant alterations over 6 months. Furthermore, the filler promoted periosteal angiogenesis, induced periosteal thickening to reinforce deep tissue support, and stimulated regeneration of dermal collagen and elastic fibers. Extended observation of two PVA/HA filler groups over 12 months showed equivalent results (Figure S1A,B show the ultrasound images of the parietal bone and the H&E‐stained sections of the dorsal region 12 months after the filler implantation). The PVA/HA filler containing triple‐dose microspheres was injected subcutaneously into rats to evaluate the associated inflammatory response, and the inflammatory level remained stable at the 12‐month time point (Figure S1C illustrates the inflammatory levels observed 12 months following the subcutaneous implantation of the filler). Collectively, the filler maintained mild inflammatory responses while preserving its capacity for enhancing periosteal thickening and collagen fiber proliferation.

In this study, the novel filler composed of PVA and HA effectively integrated the benefits of its individual components. This resulted in enhanced mechanical strength and stability of the gel while significantly reducing the biodegradation rate of the composite filler and extending its longevity. Distinct from CaHA, PVA does not release calcium or phosphate ions, thus avoiding adverse effects on osteoblast proliferation during localized high‐density administration [19]. Compared with PMMA and PLLA, it may be less irritating, cause less inflammation, and have higher safety [20]. Furthermore, the material properties of the PVA microspheres also draw on those of natural cartilage, simulating a favorable biochemical environment and thereby providing a foundation for the growth and differentiation of chondrocytes [21]. Regarding biocompatibility, the PVA/HA filler also promoted the repair and regeneration of fibrillary connective tissue [22].

In general, microspheres with diameters over 20 μm are not typically engulfed by macrophages, thereby reducing the occurrence of inflammation and granuloma formation [23]. In this study, the PVA microspheres had diameters larger than 20 μm, and following implantation in both groups of rats, there were no signs of acute or chronic inflammatory reactions, foreign body responses, tissue necrosis, vascular embolism, or granuloma formation after implantation in either group of rats. Considering that the HA component partially degrades after 6 months of implantation, and based on the medical consensus regarding touch‐up treatment, patients may opt for repeated implantations of this filler. This can result in a continuous increase in the accumulation of PVA microspheres within the tissue, so during the study, increase the amount of PVA microspheres. At different observational time points, H&E staining and anti‐IL‐6 immunohistochemical analyses revealed only mild inflammation, which is consistent with previous research [24]. When the experimental duration was extended to 12 months, the inflammation around the microspheres remained low. No reactive astrocytes in macrophages were observed around the PVA microspheres, indicating a low likelihood of chronic granulomatous reactions. These findings indicated that the local physical stimulation from the filler led to a mild inflammatory response, supporting the safety and biocompatibility of the gel in vivo.

The PVA/HA fillers were injected into the periosteum of rats. Over time, the number of capillaries near the periosteum increased under the influence of the filler. The capillary numbers at the periosteum of the Single PVA group and the Triple PVA group increased significantly at all time points compared to the Control group, suggesting that the PVA/HA filler may promote angiogenesis [25]. Using anti‐CD31 immunohistochemistry, significantly more capillaries were seen around the periosteum in the Single PVA group than in the Control group at three time points (Figure S2 presents the CD31 immunofluorescence images, which show capillaries in the periosteal region). The growth of the periosteum is largely affected by nutrients supplied from capillaries, promoting its continuous growth and thickening, undoubtedly providing more lasting positioning and shaping support [26]. A significant correlation was observed between periosteal thickness and parietal bone ultrasound height in the Single PVA group, indicating that the sample achieves plastic effects by promoting periosteal thickening (Figure S3 presents a scatter plot illustrating the relationship between periosteal thickness and parietal bone ultrasound height). In contrast, HA alone exhibited minimal changes in capillary formation and periosteal thickness, limiting its ability to induce deep‐seated effects. Both the Single and Triple PVA microspheres in the filler fall within the optimal range of mechanical stimulation, which is neither excessive nor insufficient, and effectively promotes the gradual thickening of the periosteum [27]. These findings underscore the potential of PVA/HA fillers as a sustainable approach for combating skin aging by leveraging their ability to enhance local vascularization and stimulate beneficial tissue remodeling.

The PVA/HA filler demonstrated sustained support, maintaining dimensional stability in both periosteum and subcutaneous tissue for at least 6 months post‐implantation. This persistence may be attributed to periosteal thickening and augmented fibroconnective tissue formation. This structural reinforcement likely results from optimized biomechanical properties through the integration of PVA and HA; the hydrogel modulates mechanical stress distribution across periosteal layers, thereby inducing deep tissue reinforcement. Furthermore, biomechanical stimuli from the filler significantly upregulated collagen deposition, elastic fiber density, and fibroblast functions, enhancing collagen biosynthesis while improving fibroblast adhesion and proliferation [28, 29]. Concurrently, material‐induced cutaneous tension promoted elastic fiber reorganization under physiological loading, leading to increased elastic fiber density in subcutaneous tissue [30].

There are some limitations to the current study. Future investigations will extend the observation period, compare the differences between the present PVA/HA sample and the commercially available PVA/HA/HPMC filler, and further elucidate the mechanism underlying the efficacy of the PVA/HA composite in stimulating periosteal growth [31]. This study could be further expanded on in more diverse animal models and clinical applications. Future studies should prioritize exploring the efficacy of this intervention in different animal models, which will facilitate the assessment of risks associated with chronic granulomas and vascular embolism, thereby enabling a more comprehensive evaluation, and ultimately translate it to human applications [32]. Additionally, future research should focus on conducting more in‐depth investigations from both materials science and clinical perspectives.

## 5 Conclusions

In conclusion, the PVA/HA filler, a Novel Cross‐Linked Sodium Hyaluronate Gel with PVA Microspheres, demonstrated strong structural support in both the periosteum and subcutaneous tissue. Preclinical findings indicated the filler's potential to enhance periosteal angiogenesis and promote periosteal thickening, while stimulating collagen fiber formation and exhibiting low inflammation, high safety, and no risk of hyperplasia.

## Author Contributions

Y.D. performed the experiment. X.L. helped perform the design of the methodology. Z.L. contributed to data curation and manuscript preparation. H.N. contributed significantly to analysis and writing – review and editing. S.W. helped perform the analysis with constructive discussions. D.T. conceived the idea and revised the manuscript. All authors approved the final version of the manuscript.

## Ethics Statement

The authors confirm that the ethical policies of the journal, as noted on the journal's author guidelines page, have been adhered to and the appropriate ethical review committee approval has been received. All animal experimental manipulations were approved by the Ethics Committee of Chongqing Medical University on 2023‐11‐23 (IACUC‐CQMU‐2023‐0379).

## Consent

Dongmei Tan hereby authorizes Journal of Cosmetic Dermatology to use the accompanying images of this article for the publication of the paper titled ‘A Novel High‐Safety Filler with Potential Periosteal Enhancement: In‐Vivo Evaluation of Cross‐Linked Sodium Hyaluronate Gel with PVA Microspheres in Rat Model’. This article does not involve clinical trials.

## Conflicts of Interest

The authors declare no conflicts of interest.

## Supporting information


**Figure S1:** The PVA/HA filler remains effective even 12 months after injection. (A) Ultrasound images of the injection sites in the periosteum and subcutaneous tissue of each group after 12 months. (B) The H&E staining images of the injection sites in the periosteum and subcutaneous tissue in each group after 12 months (100×). (C) Quantitative analysis of inflammatory responses in H&E stained sections from subcutaneous injection samples of each group over a 12‐month period showed no significant differences; *n* = 3.
**FIGURE S2:** The CD31 immunofluorescence images showing capillaries at the periosteum in each group (200×).
**FIGURE S3:** Scatter plot of periosteal thickness and parietal bone ultrasound height in the single PVA group, groups were compared using the Pearson correlation analysis with a significance threshold of *p* < 0.01.

## Data Availability

The data that support the findings of this study are available from the corresponding author upon reasonable request.
