# Supplementary material for: A Novel High‐Safety Filler with Potential Periosteal Enhancement: In Vivo Evaluation of Cross‐Linked Sodium Hyaluronate Gel with PVA Microspheres in Rat Models
Source: J Cosmet Dermatol. 2025 Oct 29;24(11):e70522. doi: 10.1111/jocd.70522 (PMC12569972; doi:10.1111/jocd.70522)
Supplement: Supplementary file 1 — Figure S1: The PVA/HA filler remains effective even 12 months after injection. (A) Ultrasound images of the injection sites in the periosteum and subcutaneous tissue of each group after 12 months. (B) The H&E staining images of the injection sites in the periosteum and subcutaneous tissue in each group after 12 months (100×). (C) Quantitative analysis of inflammatory responses in H&E stained sections from subcutaneous injection samples of each group over a 12‐month period showed no significant differences; n = 3. FIGURE S2: The CD31 immunofluorescence images showing capillaries at the periosteum in each group (200×). FIGURE S3: Scatter plot of periosteal thickness and parietal bone ultrasound height in the single PVA group, groups were compared using the Pearson correlation analysis with a significance threshold of p < 0.01. [file JOCD-24-e70522-s001.docx]

**Supporting Information**

**
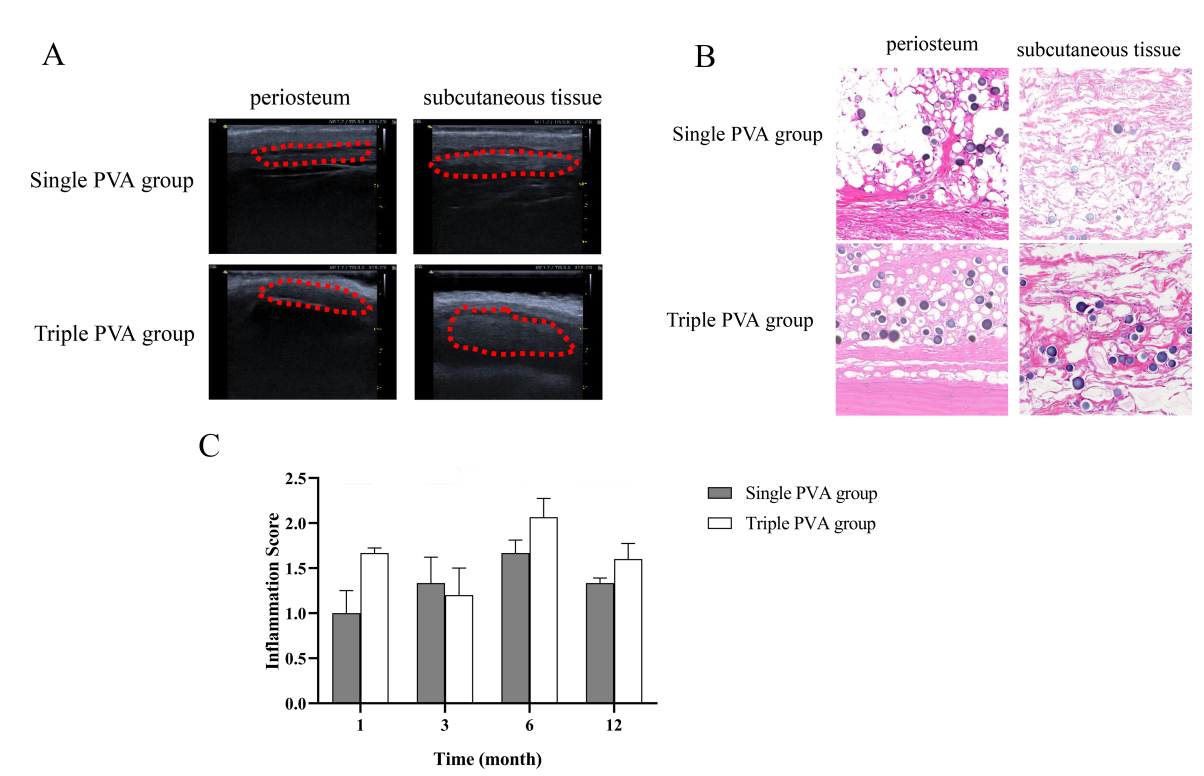
**

**Fig. S1.** The PVA/HA filler remains effective even 12 months after injection. (A) Ultrasound images of the injection sites in the periosteum and subcutaneous tissue of each group after 12 months. (B) The H＆E staining images of the injection sites in the periosteum and subcutaneous tissue in each group after 12 months (100×). (C) Quantitative analysis of inflammatory responses in H&E stained sections from subcutaneous injection samples of each group over a 12-month period showed no significant differences; n = 3.

**
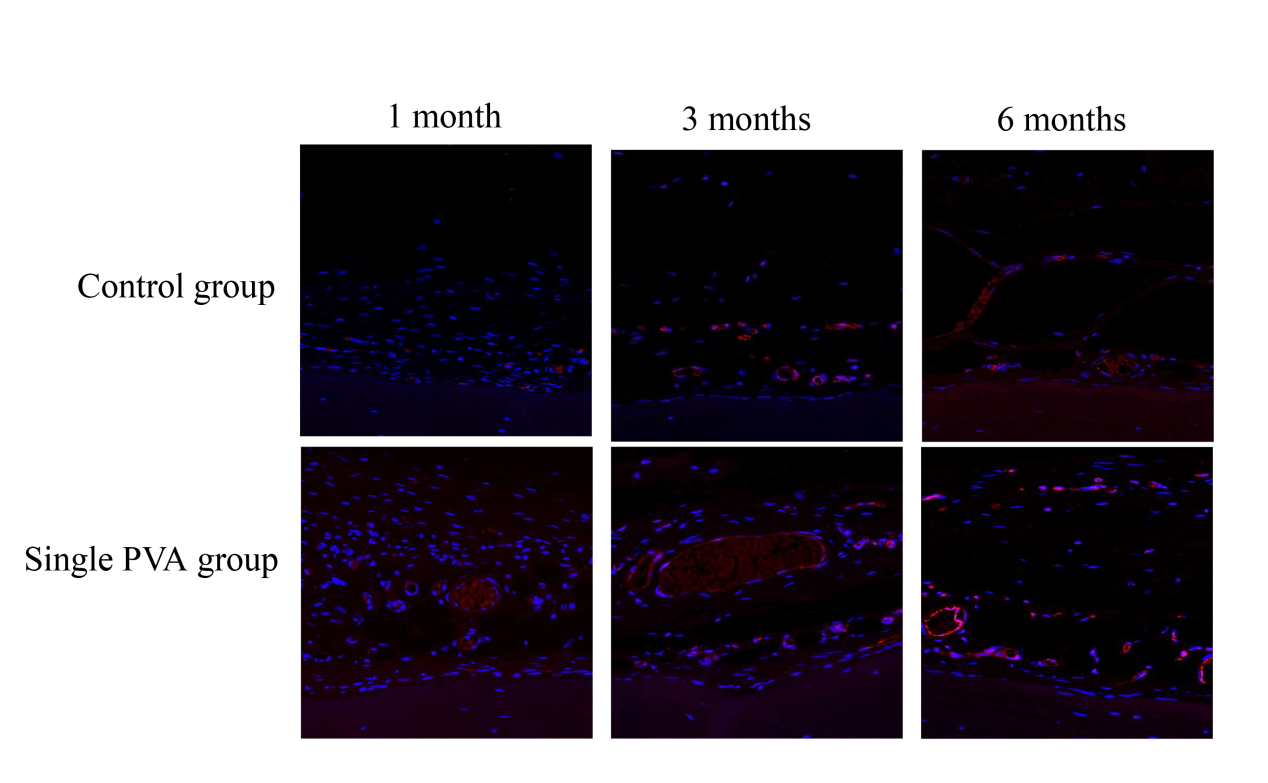
**

**Fig. S2.** The CD31 immunofluorescence images showing capillaries at the periosteum in each group (200×).


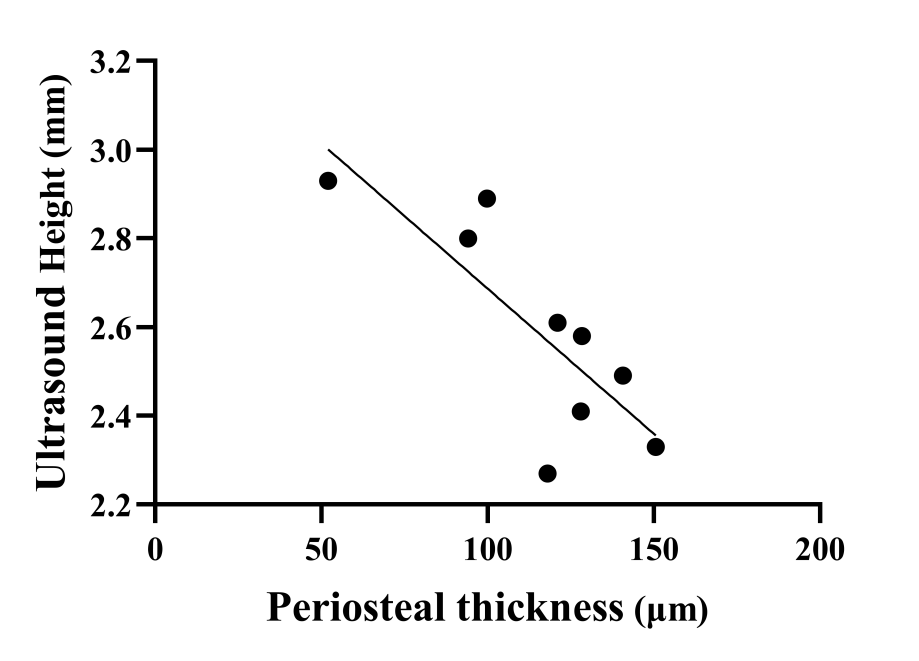


**Fig. S3.** Scatter plot of periosteal thickness and parietal bone ultrasound height in the single PVA group, groups were compared using the pearson correlation analysis with a significance threshold of *p*<0.01.
